# Interpretation of ATR-FTIR spectra of dental adhesives throughout simultaneous polymerization and solvent loss

**DOI:** 10.1371/journal.pone.0325692

**Published:** 2025-06-11

**Authors:** Arwa Almusa, António H. S. Delgado, Anne Margaret Young

**Affiliations:** 1 Division of Biomaterials and Tissue Engineering, UCL Eastman Dental Institute, London, United Kingdom; 2 Department of Restorative Dental Science, College of Dentistry, King Saud University, Riyadh, Saudi Arabia; 3 Egas Moniz Center for Interdisciplinary Research (CiiEM); Egas Moniz School of Health & Science, 2829-511 Caparica, Almada, Portugal.; Advanced Materials Technology Research Institute, National Research Centre, EGYPT

## Abstract

This study developed new Fourier transform infrared (FTIR) spectroscopy methods to assess effects of drying level on the composition and polymerization kinetics of One‐Step® (OS), Optibond^TM^ Universal (OU) and G‐Bond (GB) dental adhesives. 5 μL of each adhesive were placed in turn on an FTIR, Attenuated Total Reflectance (ATR) accessory, operating at 37ºC. Spectra were generated before, during and after light-curing (20 s, 1000 mW/cm^2^, 450−470 nm) at 10 s after placement or following 300 s of passive drying (*n* = 3). Individual spectra of solvents, monomers and fillers, combined with spectral change upon polymerization, were used to generate model spectra and quantify component levels versus time up to 300 s after start of light exposure. Polymerization rates and maximum degree of conversion were derived using a combination of polymer and monomer peaks at 1480 and 1320 cm^-1^. Inferential analyses included Kruskal-Wallis/Mann-Whitney U using a significance level of 5%. Initial acetone levels were 65, 48 and 50% in OS, OU and GB, respectively, whilst curing at 10 versus 300 s gave final acetone levels of 35, 20 and 32% versus 0, 0 and 10%. With earlier light exposure, monomer reaction rate was reduced but continued for longer leading to final conversions of 88, 86 and 40% versus 61, 66 and 77% for OS, OU and GB, respectively. The FTIR techniques developed could monitor process kinetics and demonstrate the large, highly significant effects of drying method on final polymerized dental adhesive composition and polymerization level.

## 1. Introduction

Dental adhesives are polymerizable fluids that allow a conservative approach to restoration of caries-affected teeth, by enabling restorations with resin composites [1]. Bonding to acid-etched enamel using a hydrophobic resin is generally reproducible. However, the bond with the more variable, underlying dentine and its organic constituents is much less reliable and thus requires a specific adhesive strategy. The adhesive must bond effectively, by infiltrating the pits and pores in acid-etched enamel or envelop the exposed collagen in demineralized dentine, resist shrinkage stresses that occur as the restorative filling sets, and ultimately provide long-term sealing. Furthermore, adhesives should be resistant to hydrolytic degradation, enzymatic integrity challenges and prevent continuing or recurrent decay at the tooth/restoration interface [2–7].

Commercial dental adhesives typically contain various methacrylate monomers, solvents and, in some cases, inorganic filler particles [8,9]. However, the number available in the market is vast, as are the differences in their component chemistries and ratios [1,8,10–13]. Solvents added are generally ethanol, acetone, water or a co-mixture of these [14]. These lower the adhesive viscosity and improve wetting of the hydrophilic dental tissue. During clinical adhesive placement, evaporation of these solvents is required. Under or over evaporating the solvents can strongly affect monomer polymerization kinetics [15]. Previous authors have reported that a critical solvent concentration, at which monomer mobility is counter-balanced by a detrimental effect on mechanical properties, may exist. Moreover, liquid mixtures with too low viscosity could have a delayed onset of the gel point. At this stage of polymerization, reaction rate can increase exponentially due to reduction in diffusion controlled free radical termination steps [16,17]. Decreased solvent retention in adhesives has also been confirmed to result in high bond strengths, based on meta-analytical data [18]. This proves the impact of poor solvent control on the creation of adhesive layers and their mechanical properties. Treatment success can thus be both case and operator dependent [19–22].

Most studies thus far that investigated solvent evaporation effects, have focused on determining the polymerization extents, bond strengths, solvent type and solvent evaporation methods [21,23,24]. In a previous recent study, it was shown that ATR-FTIR is a simple and sensitive method to assess varying chemistries encountered in adhesives. Furthermore, it was used to assess evaporation rates, and subsequent polymerization kinetic parameters, in acetone-based dental adhesives [10]. Varying the concentration of acetone and having it as a co-mixture with other solvents and monomers were responsible for variable results in its evaporation rates [10]. Moreover, modelling FTIR spectra has enabled determination of the composition of different types of adhesives [9], and acetone-based dental adhesives before and after solvent evaporation [9,10]. The modelling used the spectra of pure components added in varying ratios. Unfortunately, this method is invalid if evaporation and polymerization are occurring simultaneously. The work also showed, however, that difference spectra, generated by subtracting initial spectra from those post polymerization, had the same peaks and troughs irrespective of polymerization level or material. For the following work, this observation has been used to modify the modelling method [9] to enable component level determination during simultaneous polymerization and drying.

In earlier studies [9,10] changes in a monomer peak height above baseline were used to quantify polymerization extents versus time. However, with simultaneous drying and polymerization, the monomer peak can increase due to the former but also decrease due to the later process invalidating the method. To address this issue, in the following a new method has been devised to extract polymerization extents versus time. This employs both a reactant and product peak. This method is possible because the product peak, unlike the monomer peak, increases with both drying and polymerization.

The new methods will be used to enable assessment of the impact of different solvent evaporation methods on the chemistries of three different acetone-based commercial dental adhesives. The hypotheses are that level of drying of adhesives before light-activated polymerization has a measurable effect on the final FTIR spectra and final component levels. Furthermore, it affects both rates and final levels of methacrylate polymerization.

## 2. Materials and Methods

### 2.1 Materials

Components of the three investigated adhesives, One‐Step ® (Bisco Inc, Schaumburg, IL, USA), G‐Bond (GC Corporation, Tokyo, Japan), and Optibond^TM^ Universal (Kerr, Orange, CA, USA), are as disclosed on the manufacturers’ website. Their chemical structures and FTIR peak assignments were given in earlier work [10].

### 2.2 ATR-FTIR spectral acquisition

The spectra of commercial adhesives were acquired using an FTIR spectrometer (FTIR Spectrum One, PerkinElmer, Beaconsfield, UK) and an Attenuated Total Reflectance (ATR) golden gate accessory (Golden Gate ATR, Specac, Orpington, UK). The temperature was set to 37 °C utilizing a temperature controller (Specac Ltd. Orpington, UK). A micro syringe (SuperfleX™ Syringe, Sigma‐Aldrich, USA) was used to place (5 μL) of the adhesive on the ATR diamond. The adhesives were light cured using a Demi Plus (Kerr, Brea, CA, USA) light emitting diode (LED) light curing unit. The light had a wavelength between 450 nm and 470 nm and an output of 1000 mW/cm^2^ irradiance, checked with a Demetron Dental Curing Radiometer Model 100 (Demetron Research Corp., Danbury, CT, USA) analogue radiometer.

Adhesives were light cured for 20 s, either 10 s after placement, or following 300 s of passive drying at 37 °C on the ATR. Spectra were acquired every 5 s from placement, for 600 or 300 s with versus without 300 s passive drying, respectively (repetition, n = 3 per adhesive). Final spectra were first compared to determine if the adhesive or drying method had significant effects.

Spectra of solvents, pure monomers and fillers required to generate model spectra are provided in supplementary data and plotted in previous work [10]. The adhesives’ fillers were separated through multiple acetone addition/ washing, centrifugation and drying steps as previously described in detail [10]. To facilitate acquisition of filler spectra, the (ATR) golden gate sapphire anvil was utilized to ensure a close contact with the diamond. All spectra were obtained between 700 and 4000 cm^−1^ with an 8 cm^−1^ resolution and analysed via TimeBase software v. 16.0 (Perkin-Elmer, UK).

### 2.3 Difference spectra

Previous work had shown that 300 s of drying on the FTIR is sufficient for the adhesives in this study to reach a stable dried state [10]. Subtracting initial spectra from those up to 300 s was used in the following to determine absorbance change (difference spectra) due to drying only. Upon subsequent light exposure, rapid polymerization then occurs. Subtracting spectra after 300 s from those at 300 s was used to obtain difference spectra upon polymerization only. These were compared with the difference spectra upon simultaneous drying and polymerization.

### 2.4 Change in component levels over time

If drying only is occurring, reaction extents (fraction of final reaction level) can be obtained using absorbance at any wavenumber where there is significant change [10]. Drying extents were previously found to be 50% of final values after 23, 25 and 112 s for OS, OU and GB respectively. The aim of the following was to assess how light exposure at 10 s affected this process.

To quantify chemical changes upon simultaneous drying and light exposure, spectra at different time points were modelled using the spectra of the pure components and the difference spectrum obtained upon polymerization. Spectra generated at 5, 10, 15, 20, 30, 50, 100, 200 and 300 s were used to evaluate the changes in composition over time with no passive drying. Spectra at 600 s, with polymerization at 300 s, were also modelled to compare final compositions with versus without passive drying.

Model spectra were created using Microsoft Excel Tools v.16.35 (Microsoft, Redmond, WA, USA). In this method, the spectra of the pure components for each adhesive with the difference spectrum for polymerization were combined in various ratios until all the key peaks in the adhesive spectra at different times were accounted for. Further changes were then made until the model and adhesive peak heights matched as well as possible. The best fit was when the modulus of the difference between the adhesive and model absorbance, summed over all wavenumbers, was at a minimum.

Absorbance of the adhesive model (A_m,v_) at a particular wavenumber (v) is expected from the Beer Lambert law to equal to the total of the absorbances of the pure components multiplied by their relative volume fractions plus a contribution due to polymerization. Therefore:


$${A\;}_{m,v}=\;\sum \left(A_{x,v}\;F_{x}\right)+\;\left({\Delta A}_{p,v}\;F_{p}\right)\;\;$$ (1)

A_x,v_ denotes the absorbance of a pure adhesive component (x) at a given wavenumber and (F_x_) represents its volume fraction in contact with the FTIR diamond. ΔA_p,v_ is the absorbance change between 300 and 600 s upon polymerization following 300 s of passive drying of each adhesive. F_p_ is the fraction of this change occurring at different times. If the model fit is ideal, then the following are expected:


$$\sum \left(F_{x}\right)\;=\;1$$ (2)

$$\sum \left|{(A}_{m,v}\;–\;A_{a,v}\right|=\;0\;\;\;$$ (3)

A_a,v_ represents the actual adhesive’s absorbance whilst the vertical lines indicate the modulus of values. Non ideal fit occurs if there are interactions between components or the spectrum of a major component is unknown. Two monomers, glycidyl phosphate dimethacrylate (GPDM) and BPDM, were not available. To account for their peaks, other monomers, 10-methacryloyloxydecyl dihydrogen phosphate (10-MDP) and triethylene glycol dimethacrylate (TEGDMA), that had similar chemical groups were used.

### 2.5 Polymerization kinetics

The degree of monomer conversion versus time was calculated using the following equations. The fraction of the methacrylate groups polymerized is given by:


$$y=\frac{\left[P\right]}{\left[P]+[M\right]}$$ (4)

Where [] indicates molar concentrations of polymerized (P), and unpolymerized (M), methacrylate groups. From the change in spectra with time, an increase due to formation of polymer is seen at 1480 cm^-1^ associated with increasing CH_2_ bending. From the Beer Lambert law, the height of the peak at 1480 cm^-1^ at time t after the start of the light exposure above an initial level at baseline will be:


$$h_{1480}=A_{1480,t}-A_{1480,0}=k_{p}[P\;$$ (5)

While the height of the monomer peak at 1320 cm^-1^ above a background of 1335 cm^-1^ is:


$$h_{1320}=A_{1320,t}-A_{1335,t}=k_{m}[M\;\;\;\;\;\;\;\;\;\;\;\;\;\;\;\;\;\;\;\;\;\;\;\;\;\;\;\;\;\;\;$$ (6)

A_v,0_ and A_v,t_ are the adhesive absorbance at wavenumber, v, initially and at time t. k_p_ and k_m_ are constants. Equation 6 has been used alone to determine rates of monomer conversion but only when the total concentration of methacrylates in monomer and polymer form is constant [25]. Alternatively, if this is not true, by combining equations 4, 5 and 6, fractional monomer conversion can instead be given by the following equation:


$$y=\frac{h_{1480}}{h_{1480}+\frac{k_{p}}{k_{m}}h_{1320}}\;\;\;\;\;\;\;\;\;\;\;\;\;\;\;\;\;\;\;\;\;\;\;\;\;\;\;\;\;\;\;\;\;\;\;\;\;\;\;\;\;\;\;\;\;\;\;\;\;\;\;\;\;\;\;$$ (7)

From equations 5 and 6:


$$\frac{k_{p}\Delta [P]}{k_{m}\Delta [M]}=\frac{{\Delta h}_{1480}}{{\Delta h}_{1320}}$$ (8)

Δ indicates change in concentration or peak height above baseline. With polymerization only occurring


−Δ[M]=Δ[P] (9)

Combining 8 and 9 then leads to


$$\frac{k_{p}}{k_{m}}=\frac{{-\;\Delta h}_{1480}}{{\Delta h}_{1320}}\;\;\;\;\;\;\;\;\;\;\;\;\;\;\;\;\;\;\;\;\;\;\;\;\;\;\;\;\;\;\;\;\;\;\;\;\;\;\;\;\;\;\;\;\;\;\;\;\;\;\;\;\;\;\;\;\;\;\;\;\;\;\;\;\;\;\;$$ (10)

For a polymerizing methacrylate, k_p_/k_m_ should be constant. In this study, when polymerization only was occurring for passive dried samples for the bisphenol A-glycidyl methacrylate (Bis-GMA) based adhesives OS and OU this ratio was 1.03 and 1.02 respectively. Conversely, for the urethane dimethacrylate (UDMA) based adhesive GB, this ratio declined to 0.71. These k_p_/k_m_ values were therefore used with equation (7) to estimate conversion versus time. Previous studies showed k_m_ was 14 and 20 mol^-1^ cc^-1^ of methacrylate groups for pure BIS-GMA versus UDMA (25). Multiplying these by k_p_/k_m_ gives k_p_ equal to 14 mol^-1^ cc^-1^ irrespective of the monomer increasing confidence in the method.

The extrapolated final degree of conversion (D_C,max_) was found by determining the y axis intercept of late time conversion versus inverse of time. To determine the rate of the polymerization reaction, the gradient of a tangent of the conversion curve during the 20 s duration of light curing was calculated.

### 2.6 Statistical analysis

Statistical analysis was performed using the Statistical Package for the Social Sciences software SPSS version 28.0 for Mac (IBM, New York, USA). The level of significance was set at 5%. For this set of data, Shapiro-Wilk test revealed that the data were not normally distributed (*p* < 0.05). Therefore, non-parametric tests Kruskal-Wallis and Mann-Whitney U were employed for inferential analysis.

## 3. Results

### 3.1 Final spectra with versus without passive drying

**Fig 1:**
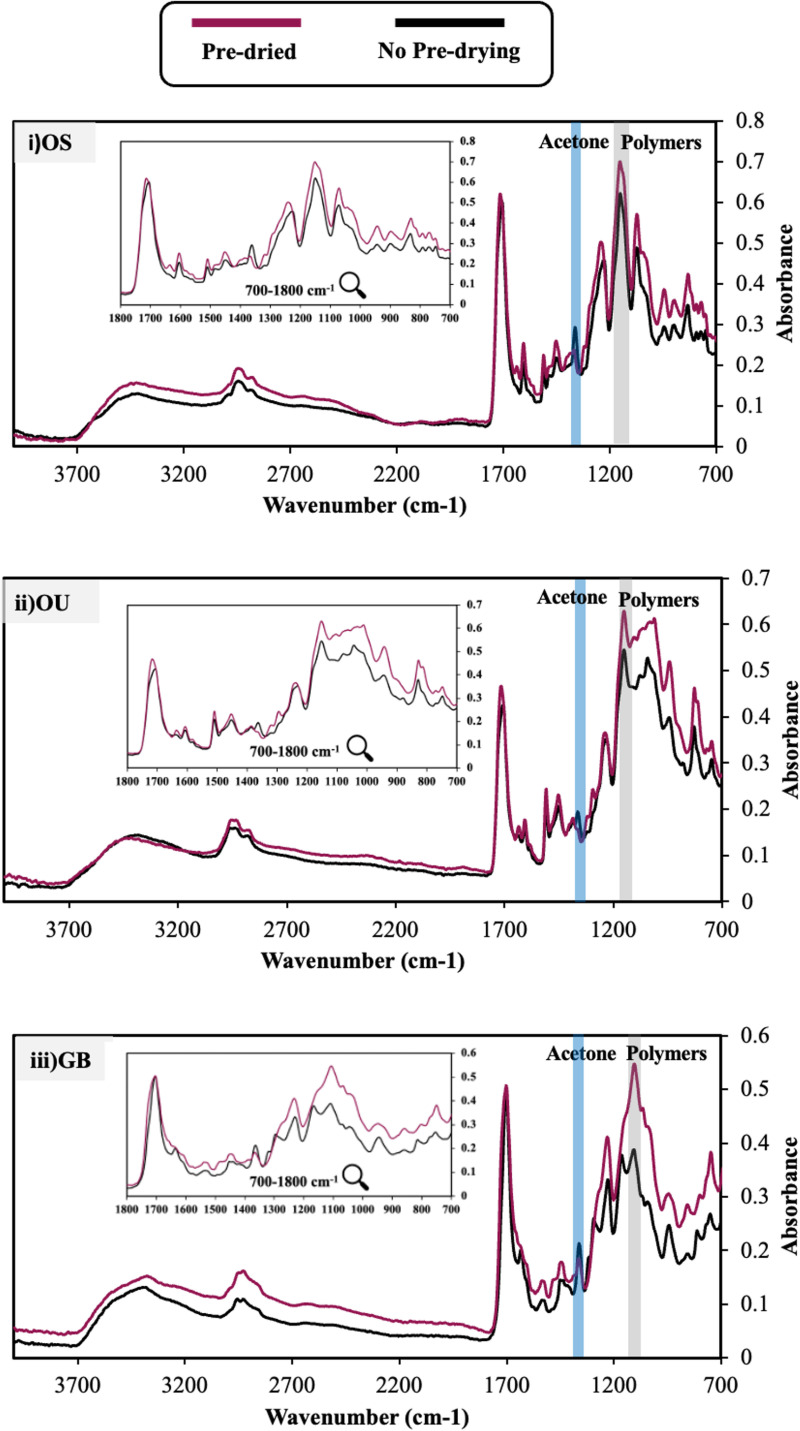
Final average spectra of adhesives. Final average (n = 3) FTIR spectra of **i)** OS, **ii)** OU and **iii)** GB. Black spectra are at 300 s, following light cure for 20 s, without passive drying. Red spectra are at 600 s following passive drying for 300 s then light curing for 20 s. Acetone peaks at 1360 cm^-1^ are highlighted via a blue bar and monomer/ polymer peaks at 1150 cm^-1^ are highlighted via a grey bar.

Fig 1 shows the average (*n* = 3), final FTIR spectra of the adhesives, 300 s after light exposure, with versus without 300 s of passive drying. Raw data are in supplementary spreadsheets. Differences are mainly seen in the fingerprint (1200–700 cm^-1^) region. Average absorbance at 1150 cm^-1^ (due to monomers, polymers and filler) and at the acetone peak at 1360 cm^−1^ are provided in Table 1. Without passive drying, for all three adhesives the final 1150 cm^-1^ absorbances were significantly reduced and acetone peaks higher (Mann-Whitney U, *p* < 0.05) consistent with reduced drying level. Additionally, with OS and OU, methacrylate peaks at 1640 (C = C stretch) and 1320/ 1295 cm^-1^ (C-O stretch) were less obvious. This could be due to decreased solvent evaporation and/ or increased polymerization fraction. Conversely, with GB, these methacrylate peaks were more visible without passive drying indicating reduced polymerization.

**Table 1:** Absorbance at 1150 cm^-1^ and 1360 cm^-1^ for OS, OU and GB with and without passive drying [standard deviation] (n = 3).

Adhesive	Peak absorbance (1150 cm^ − 1^)		Peak absorbance (1360 cm^-1^)	
	No passive drying	Passive dried	No passive drying	Passive dried
OS	0.621 [0.007] ^A^	0.700 [0.009] ^B^	0.291 [0.003] ^A^	0.216 [0.003] ^B^
OU	0.541 [0.044] ^A^	0.625 [0.003] ^B^	0.191 [0.020] ^A^	0.150 [0.001] ^B^
GB	0.346 [0.015] ^A^	0.452 [0.019] ^B^	0.208 [0.013] ^A^	0.176 [0.002] ^B^

Different superscript capital letters indicate a statistically significant difference between the passive dried and not dried group for a specific adhesive (Mann-Whitney U, p < 0.05).

### 3.2 Difference spectra upon separate drying and polymerization or both simultaneously

**Fig 2:**
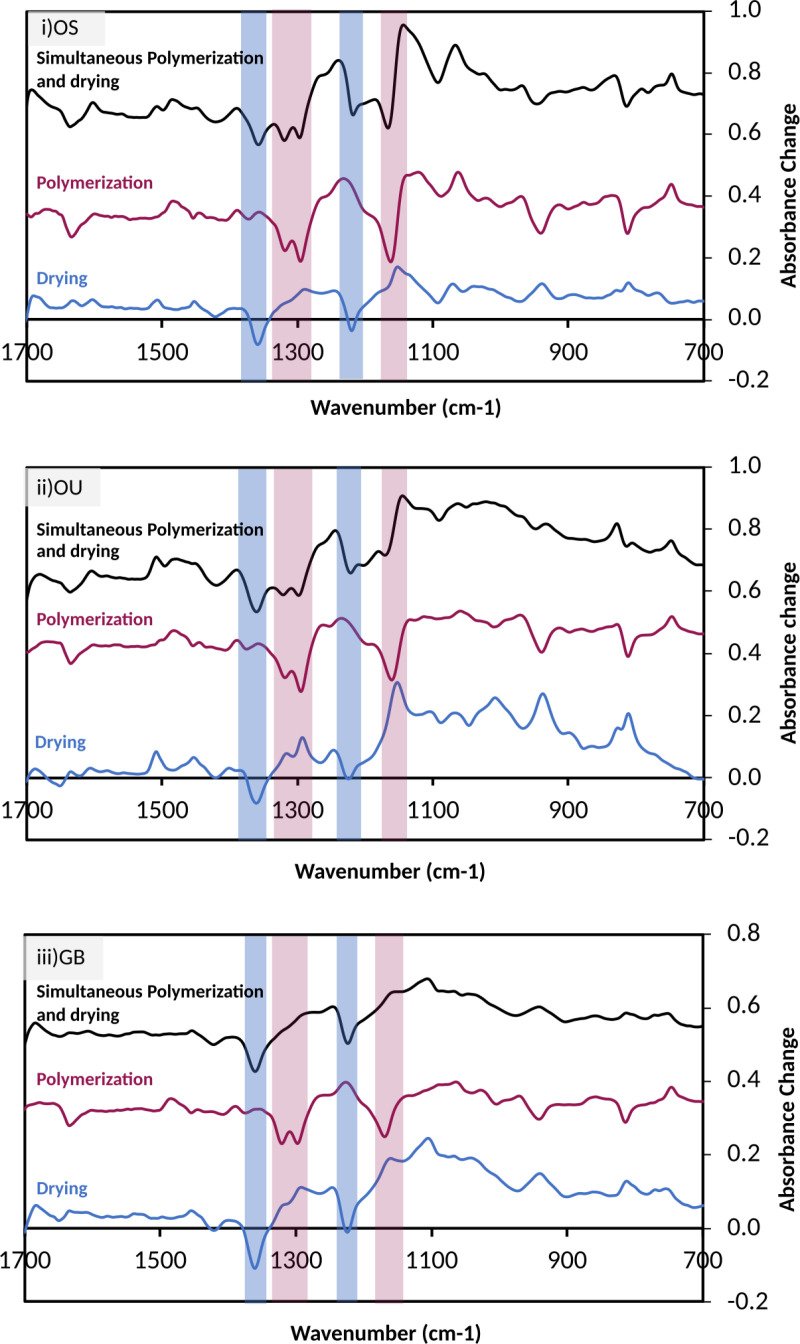
FTIR difference spectra of adhesives. FTIR difference spectra for **i)** OS, **ii)** OU and **iii)** GB (n = 3). Those upon drying-only, between 20 and 300 s, are provided in blue. Polymerization after drying, from 300 to 600 s with light exposure from 300 to 320 s, are given in red. Spectra in black are spectral change between 10 to 300 s with light exposure at 10 to 30 s and no passive drying. Bars highlight the main troughs caused by reduction in absorbance, upon drying, due to acetone evaporation (blue) and monomer polymerization (red).

To quantify chemical changes, difference spectra for separate drying and polymerization are first required. Example averaged difference spectra for separate drying and polymerization steps for each adhesive are shown in Fig 2 in blue and red, respectively (raw data are in supplementary spreadsheets). Drying-only FTIR difference spectra were generated by subtracting any spectrum up to 300 s in the passive dried groups, from their initial spectra. These had peak and trough positions that were adhesive (compare blue spectra in Fig 2), but less time dependent. This was consistent with different adhesive compositions but for a given adhesive, the same process irrespective of time.

Polymerization only difference spectra were obtained by subtracting spectra after 300 s from those at 300 s (just before the onset of polymerization) in the passive dried group. These had peak and trough positions that were less adhesive sensitive (compare red spectra in Fig 2). This is expected if the same change (i.e., methacrylate group reaction) is occurring irrespective of the adhesive. The level of change is proportional to the concentration of methacrylate groups that have polymerized. The final level of change upon polymerization with GB was less than that of OS and OU suggesting GB has lower total methacrylate group concentrations and/ or a reduced polymerization fraction.

The main difference spectra troughs for drying, at 1220 and 1360 cm^−1^, were due to acetone evaporating. The main troughs for polymerization were at 1150 cm^-1^ [v(C-O)], 1300 and 1320 cm^−1^ [v(C-O)] and at 1640 cm^−1^ [v(C = C)]. These are characteristically observed upon methacrylate polymerization with resin-based dental materials. Peaks at these wavenumbers are seen in the drying difference spectra due to monomers concentrating, as opposed to polymerizing. Both polymerization and drying caused increase in absorbance in the fingerprint region below 1150 cm^-1^. Only polymerization gave a peak at 1480 cm^-1^ (CH_2_ bend).

Average change in absorbance versus wavenumber between 20 and 300 s for each adhesive light-cured without passive drying are provided in black in Fig 2. In this case, difference spectra for OS and OU show characteristic troughs for both drying and the polymerization reaction. However, GB shows mainly troughs associated with drying. To quantify the chemical changes spectra modelling is required.

### 3.3 Change in component levels with time

Model FTIR spectra obtained using equation 1 of OS and OU gave generally good agreement with actual adhesive spectra versus time. Conversely for GB they were less able to identify all peaks (examples of fitting spreadsheets are provided in supplementary data). Initial and final component levels upon passive drying then polymerization, calculated by modelling are provided in Table 2. This gave initial acetone levels as 65, 48 and 50% for OS, OU and GB respectively whilst final levels were 0% for OS and OU but 10% for GB. Final model water and ethanol levels were also smaller than initial levels whilst most monomer and filler concentrations were generally raised.

**Table 2:** Percentages of components and polymerization difference used to generate adhesive model spectra.

Adhesive	Components/Sums	Model components’ percentages %		
		Initial	5 min after polymerization	
			No drying	Passive dry
OS	Bis-GMA	21 [2]	33 [3]^A^	56 [1]^B^
	TEGDMA (BPDM)	(12) [0]	(27) [3] ^A^	(28) [3] ^A^
	HEMA	13 [3]	37 [3] ^A^	32 [3] ^A^
	Acetone	65 [0]	35 [0] ^A^	0[0] ^B^
	**Sum of percentages**	111	132	116
	**Polymerization difference spectrum**	0 [0]	100 [0]	100 [0]
	**Sum of modulus of difference**	22 [3]	36 [6]	40 [1]
OU	Bis-GMA	13 [2]	33 [3] ^A^	40 [0]^B^
	GDMA	12 [2]	30 [3] ^A^	40 [0] ^B^
	HEMA	5 [0]	5 [0] ^A^	0 [0] ^B^
	10-MDP (GPDM)	(8) [0]	(10) [0] ^A^	(10) [0] ^A^
	Water	13 [2]	8 [2] ^A^	5 [0] ^B^
	Ethanol	5 [0]	5 [0] ^A^	0 [0] ^B^
	Acetone	48 [0]	20 [0] ^A^	0 [0] ^B^
	Filler	5 [0]	12 [1] ^A^	35 [0] ^B^
	**Polymerization difference spectrum**	0 [0]	95 [5]	100 [0]
	**Sum of percentages**	109	123	130
	**Sum of modulus of difference**	20 [1]	19 [6]	21 [0.3]
GB	UDMA	9 [1]	14 [2] ^A^	15 [0] ^A^
	TEGDMA	22 [3]	40 [2] ^A^	35 [0] ^B^
	10-MDP	2 [0]	8 [0] ^A^	10 [0] ^B^
	Water	19 [1]	19 [1] ^A^	14 [1] ^B^
	Acetone	50 [0]	32 [3] ^A^	10 [0] ^B^
	Filler	3 [0]	4 [0] ^A^	15 [4] ^B^
	**Polymerization difference spectrum**	0 [0]	50 [1]	100 [0]
	**Sum of percentages**	105	117	99
	**Sum of modulus of difference**	34 [3]	38 [6]	35 [0.5]

Results are provided initially and 300 s after polymerization with versus without passive drying [standard deviation] (n = 3). Different superscript capital letters indicate a statistically significant difference with versus without passive drying (Mann-Whitney U, p < 0.05). Modulus of difference indicates the accuracy of the model fit. 10-MDP and TEGDMA were used as a model for GPDM and BPDM, respectively.

**Fig 3:**
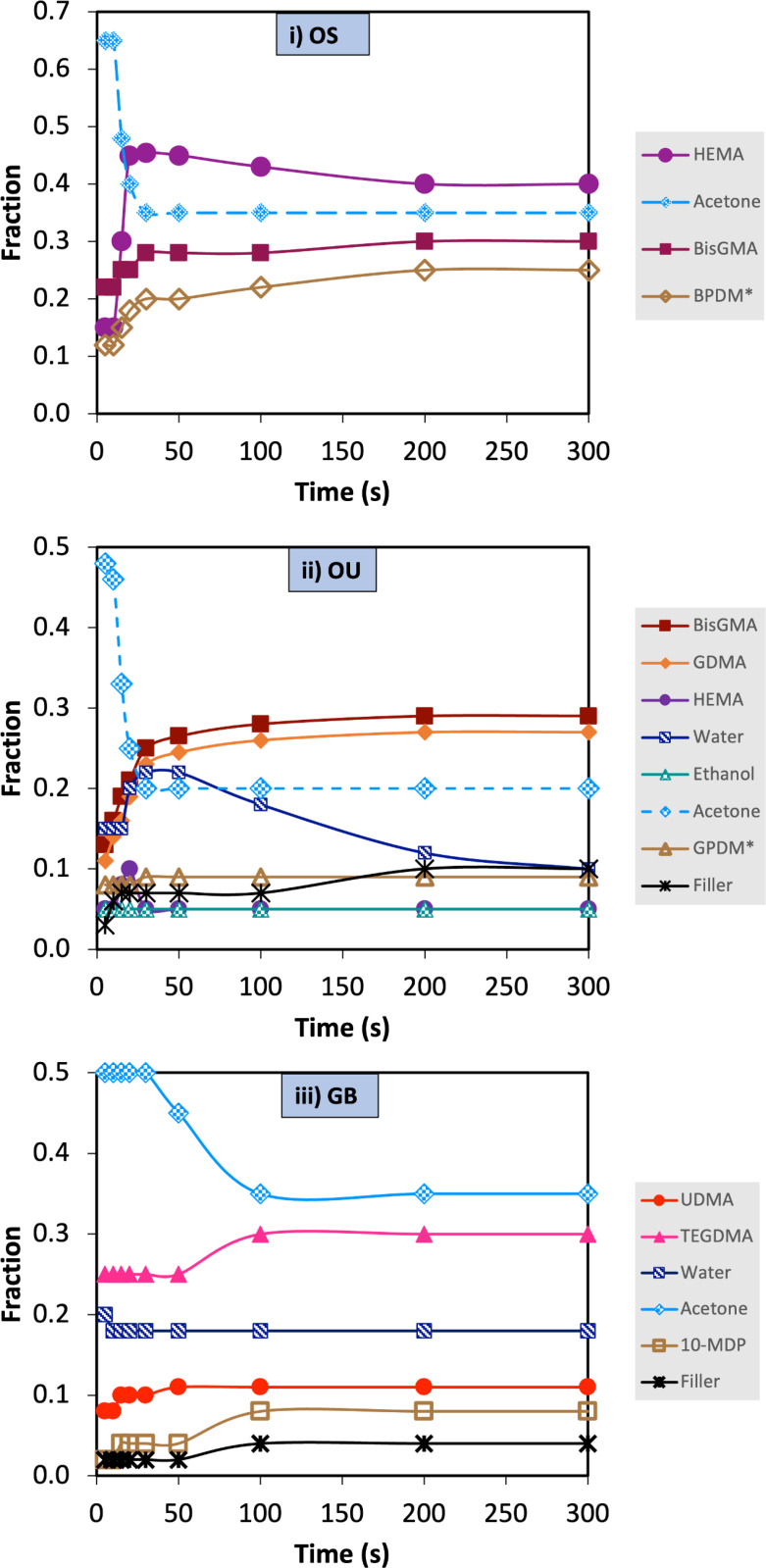
Polymerizing monomer fractions and solvent versus time. Polymerizing monomer fractions and solvent versus time (light curing from 10 to 30 s) without passive drying determined using equation 1 for **i)** OS, **ii)** OU and **iii)** GB. (*) indicates where TEGDMA and 10-MDP spectra were used as a model for BPDM and GPDM, respectively. During periods of rapid change, modelling indicated that the monomers were increasingly in polymerized form.

Upon light exposure with no passive drying, modelling showed OS and OU acetone level reduced then levelled at final values of 35 and 20%, respectively by 30 s (see Fig 3 and Table 2). With OU, water content increased during acetone loss then exhibited a slow decline. With GB there was a delay of 30 s before any acetone loss. This was followed by a slow decline in acetone content before levelling at 32% at 100 s. All model final solvent levels (acetone, ethanol and water) were significantly higher with the passive drying stage removed (p < 0.05). Final levels of BisGMA in OS and OU were also significantly lower without the passive drying stage whilst the HEMA contents were similar or raised. In OU and GB, final filler contents were also significantly reduced upon removal of the passive drying stage (p < 0.05).

### 3.4 Polymerization kinetics

Modelling using equation 1 indicated that with OS and OU there were similar levels of methacrylate groups polymerizing with versus without passive drying (see polymerization difference spectrum percentages in Table 2). As the monomer concentrations are lower with no passive drying this suggests a potentially higher monomer conversion fraction. Conversely with GB there was less methacrylate reaction with no passive drying suggesting reduced monomer conversion fractions.

**Fig 4:**
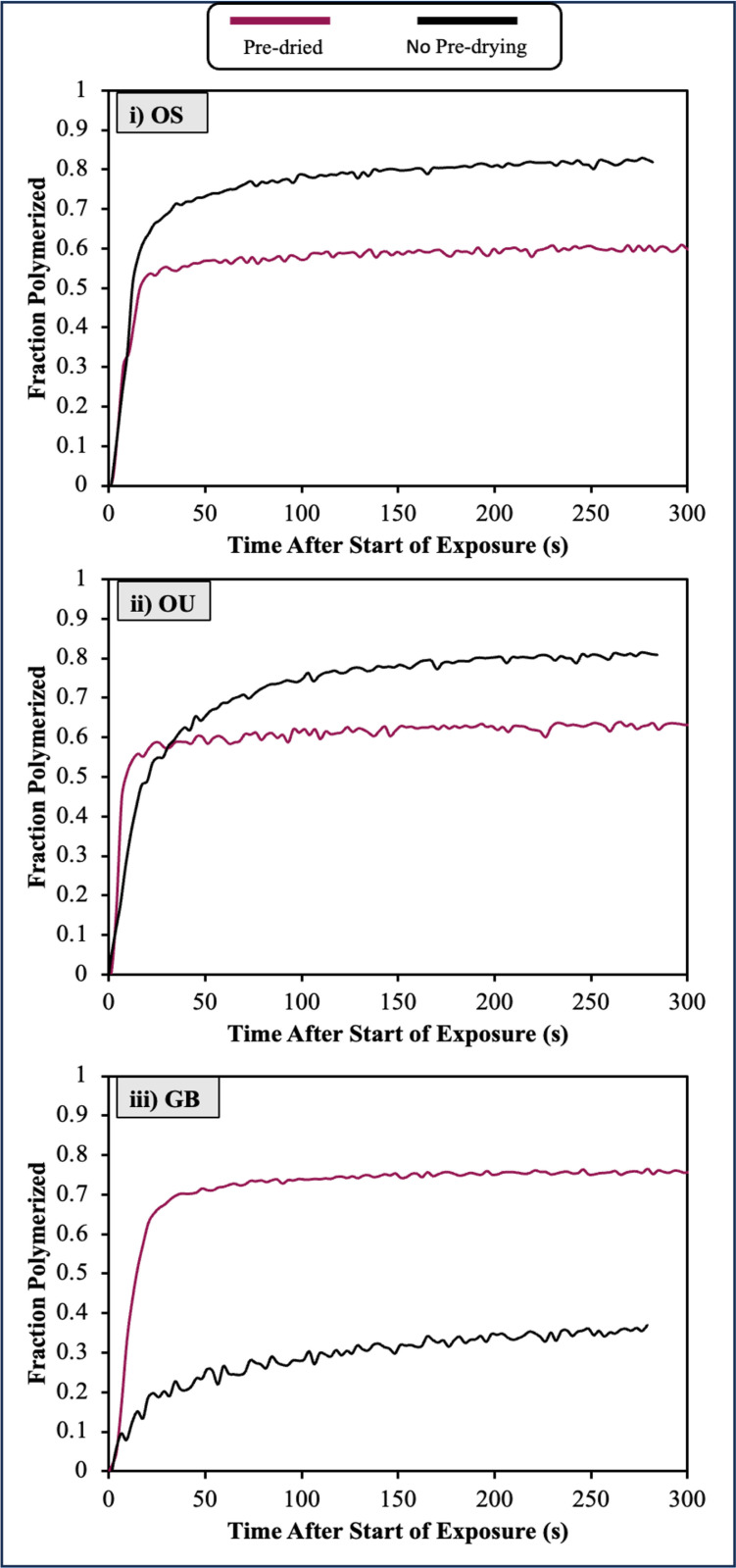
Average final degree of conversion. Average final degree of conversion versus time either passive dried for 300 s (red) or not passive dried (black) for **i)** OS, **ii)** OU and **iii)** GB (n = 3).

To determine actual polymerization fractions versus time, equation 7 was used. Results are provided in Fig 4 whilst initial rates of reaction after the start of light exposure and extrapolated final conversions D_C,max_ are given in Table 3. Example calculations are provided in supplementary spreadsheets. The early rates of polymerization seen during light exposure were between 4.8 and 6.8% s^-1^ with passive drying but substantially reduced without (0.7 to 3.1% s^-1^). Rates with GB were lowest in both cases. With passive dried samples, however, reaction had slowed substantially when the light was turned off. Conversely, with no passive drying, reaction continued throughout the observation period. The extrapolated final monomer conversions at infinite time for OS and OU increased from 61 and 66% to 88 and 86% upon removal of the passive drying stage. Conversely for GB final conversion decreased from 77 down to 40% with no passive drying.

**Table 3:** Extrapolated final degrees of monomer conversion and maximum rates of polymerization.

Adhesive	D_C,max_ [%]		Early rate of polymerization [% s^-1^]	
**No passive drying**	**Passive dried**	**No passive drying**	**Passive dried**
**OS**	88 [1] ^a,A^	61 [1] ^b,B^	3.1 [0.2] ^a,A^	5.9 [0.1] ^b,A^
**OU**	86 [9] ^a,A^	66 [2] ^b,B^	1.6 [0.5] ^b,B^	6.8 [0.3] ^b,B^
**GB**	40 [3] ^b,B^	77 [2] ^c,C^	0.7 [0.1] ^c,C^	4.8 [0.3] ^c,C^

*Results are e*ither with or without a period of passive drying [standard deviation] (n = 3). Different superscript small and capital letters indicate a statistically significant difference with drying level or between adhesives, respectively (Mann-whitney U, p < 0.05).

## 4. Discussion

Adhesive manufacturers strive to simplify usage by reducing application steps, often merging components into a single bottle. This study, which builds on previous work [9,10], examines three single-bottle adhesives. These were chosen since they represent the three adhesive strategies that are currently clinically available. Two of them represent the classical adhesive strategies: etch-and-rinse and self-etch, while the third one is a universal, multi-modal adhesive. Also, these adhesives were chosen since they use acetone as a solvent, which is commonly used by manufacturers in single-component adhesive formulations.

### 4.1 Justification

The present study is a novel extension to recent work [9,10] on spectral modelling of ATR-FTIR dental composite and adhesive data. The ATR-FTIR technique has many benefits including high reproducibility with minimal sample amounts, rapid data acquisition and ease of use with no sample preparation requirement. This study is follow-up research to work previously published [10] with the same adhesives. It provides new insight into how two different drying methods, can cause large changes in material composition and polymerization kinetics behaviour. These material properties are fundamental to predict the clinical behaviour and service time of dental adhesives in the oral environment, since adhesives are subject to different degrees of technique sensitivity and handling by clinicians. This results in high variability of their performance [6,26]. Use of passive drying for 5 minutes versus no drying was chosen to demonstrate the range of results that might be obtained.

In this study the volume of material applied was controlled to gain reproducible evaporation rates. This was previously shown to enable reproducible results and provide understanding of how chemical interactions affect acetone evaporation [10]. It had been suggested that inefficient drying compromises the physical properties of the final polymer, since monomers cannot effectively co-polymerize due to their spatial distance and the fact that they are plasticized [27]. In this study, however, reduced drying decreased conversion with GB but increased it with OS and OU. Additionally, solvent retention, such as leaving residual water in the adhesive layer bonded at the interface, increases its hydrolytic instability, degradation potential and may also cause phase separation of the hydrophobic/hydrophilic components [28]. Changes in the ratios of the different monomers seen above could indicate some phase separation.

### 4.2 Spectral analysis

The FTIR method provides the spectrum of the lower few microns of the sample. Passive drying of adhesives prior to polymerization produced different final spectra for all the adhesives examined. A high level of reproducibility was observed, particularly for the adhesives that underwent passive drying, as evidenced by the standard deviations in Table 1 being less than 5% of the values. Acetone was the primary solvent in all the adhesives investigated. This compound exhibits three strong peaks at 1700, 1360, and 1220 cm^-1^, attributed to C = O stretching, CH_3_ bending, and C-C-C stretching vibrations, respectively. The 1360 cm^-1^ peak is the most distinctive of the three since overlap with peaks related to other molecules present in the adhesive mixture is limited [10]. Additionally, this study showed minimal absorbance change at 1360 cm^-1^ upon polymerization. Consequently, this peak was particularly useful in determining if there are differences in levels of acetone present in passive dried versus non-passive dried adhesives. The adhesives that were not passive dried prior to polymerization had significantly higher absorbance at 1360 cm^-1^. This finding is consistent with prior research indicating that adhesives that were not dried for a sufficient duration have a higher solvent content, as determined by gravimetric analysis [19,23,29]. Converting these absorbance values to acetone content, however, requires modelling as the peak baseline is difficult to define due to the proximity of peaks due to other adhesive components.

Moreover, passive drying resulted in an increased final absorbance in the fingerprint region (between 1200−700 cm^-1^) when compared to non-passive dried adhesives. This may be due to methacrylate C-O peaks that shift to lower wavenumbers upon polymerization, polymer chain CH_2_ groups and/ or increased filler absorbance. Variations in the final methacrylate peaks at 1640 (C = C stretch) and 1320/ 1295 cm^-1^ (C-O stretch) also suggested changes in monomer conversion. As, however, monomer peaks are increased upon drying and decreased upon polymerization, new methods to quantify relative monomer versus polymer levels were required.

### 4.3 Difference spectra

Difference spectra are useful for assessing chemical reactions as they enable separation of any peak shifts associated with the reaction from peaks arising from components or parts of molecules not involved in the process [9,10]. As the chemical reaction is the same, difference spectra due to polymerization of methacrylates in different adhesives or composites are largely similar [10]. The main polymerization difference spectra troughs at 1150 cm^-1^ [v(C-O)], 1300 and 1320 cm^−1^ [v(C-O)] are caused by these peaks shifting to slightly lower wavenumbers when the methacrylate C-O becomes attached to a C-C group instead of a C = C. This process causes peaks in difference spectra adjacent to these troughs. The difference spectra peak at 1480 cm^-1^ is due to a bending vibration of CH_2_ groups in the polymer backbone that is commonly observed in hydrocarbon polymers. This is a useful peak for assessing polymer content because the other adhesive components have no peak at this wavenumber [10].

During drying, however, as adhesives contain different monomers and solvents that are all varying, difference spectra are adhesive dependent. Difference spectra with simultaneous polymerization and drying are further complicated by the possibility of polymerization induced phase separation. For this case, difficulties in FTIR interpretation in this study were successfully overcome by combining a previous modelling method with the difference spectra observed upon adhesive polymerization.

### 4.4 Spectra modelling and component levels

FTIR spectra of monomers are largely similar and dominated by peaks due to the methacrylate groups. Typically, only a small number of peaks due to other functional groups are observed. For example, BISGMA has distinctive additional peaks at 1610, 1506 and 1246 cm^-1^ whilst UDMA has strong unique peaks at 1518, 1236 and 1150 cm^-1^. These enable determination of their levels through modelling with relative ease. HEMA can be identified by its broad OH band at 3400 cm^-1^ whilst phosphate containing monomers will give a broad band around 1020 cm^-1^. A beneficial consequence of the similarities in methacrylate spectra is that a good fit of the model is possible upon using for example TEGDMA in place of BPDM or 10 MDP to account for the phosphate absorbance of GPDM. The downside, however, is that it can be difficult to differentiate whether peaks are due for example to HEMA or GDMA as both only have methacrylate and OH functional groups.

The final component levels obtained on modelling data after both passive drying and polymerization with OS are within 4% of those seen previously (10) after just the drying step. With OU, 10 MDP was previously estimated at 25% whilst with GB TEGDMA was given as 35% (10). These are both higher than seen in this study. A possible explanation is the polymerization step causing some phase separation of these components. All other components levels for OU and GB in this study were within 10% of those seen before polymerization previously (10) providing confidence in the new methodologies.

Modelling of GB may have given poorer fits than with OS and OU due to strong interactions between UDMA NH and acetone C = O groups as previously discussed [10]. For all adhesives, model fitting showed highly significant differences in final composition of adhesives with versus without passive drying before light exposure. In adhesives that were not passive dried, the final concentrations of solvents were significantly higher compared to those in passive dried adhesives. With OS and OU, light exposure without passive drying led to rapid polymerization and simultaneous reduction in acetone level. This might be explained by phase separation due to the reduction in miscibility of monomers with other components when they polymerize. Following phase separation, acetone that moves to the top surface will evaporate due to its low boiling point. The results suggest, however, that at a critical level of polymerization acetone is trapped. A possible explanation is that at this point the adhesive layer in contact with the FTIR diamond reaches a glass transition temperature and solidifies.

Final levels of BisGMA in OS and OU being significantly lower without the passive drying stage whilst the HEMA contents were similar or raised suggests some of the BISGMA may phase separate with the acetone then form a top surface layer. The spectra obtained would not detect this phase separated BisGMA due to the method only analysing the material in direct contact with the FTIR diamond. With OU, the longer-term slow decline in the water content seen in Fig 3 may be due to it phase separating then evaporating during post irradiation polymerization. Water would evaporate more slowly than acetone due to its higher boiling point. Lack of complete water loss aligns with results from previous studies, which have also shown the challenge of completely removing water from dental adhesives [10,19,30]. Further work is required for greater understanding of factors controlling phase separation in these adhesives.

In the case of GB, previous studies have shown time of half the acetone loss is more than 4 times longer than seen with OS and OU [10]. The slow evaporation of acetone is consistent with a reversible condensation reaction between secondary amino groups in UDMA and the C = O group in acetone that results in formation of an unstable enamine [10]. It is possible that this, in combination with low polymerization, further inhibits early acetone evaporation. Over longer term, however, continuing slow polymerization results in the acetone phase separating and evaporating during the post irradiation period. This process may halt when the combination of the enamine formation and low level polymerization traps all remaining acetone and inhibits further polymerization.

### 4.5 Polymerization kinetics

Conventionally, for most studies in the dental literature, the 1640 cm^-1^ peak of the methacrylate C = C bond, referenced against an internal standard at 1610 cm^-1^, is utilized for assessing post-irradiation polymerization. However, one of the issues with this method are the complications that arise due to the overlapping absorption of O-H bond vibrations in this spectral region, potentially influencing the accuracy of polymerization calculations in formulations containing water [9]. Furthermore, while the aromatic 1610 cm^-1^ C = C peak is applicable for BisGMA-based formulations, it is absent in UDMA-based formulations [31]. Hence, this study adopts the 1320 cm^-1^ C-O peak. This has been validated in various research works using baselines at either 1336 or 1350 cm^-1^, as a reliable measure of methacrylate concentration [25,32,33]. However, the proximity of 1350 cm^-1^ to the acetone peak at 1360 cm^-1^ renders it susceptible to interference from acetone evaporation. Therefore, when polymerization and evaporation occur simultaneously, the 1336 cm^-1^ is a more suitable baseline. Furthermore, the 1480 cm^-1^ (C-H) bending peak, only increased with polymer production and was not negatively affected by simultaneous solvent evaporation. Therefore, it was selected to measure polymer production by comparing it against its initial value.

Final conversions upon passive drying then polymerisation obtained previously using a single peak for OS, OU and GB were 68, 69 and 88%, respectively (10). These are within reasonable agreement with the results obtained with the new methodology. In the group that was not allowed to passive dry, OS and OU adhesives showed significantly greater conversions compared to the passive dried adhesives whilst with GB the opposite was observed. Factors controlling polymerization kinetics are complex. It has been suggested that presence of solvents decreases viscosity and increases the mobility of monomers and polymer chains, leading to higher levels of conversion [16,23,34]. Additionally, entrapped solvents within the adhesive layer may act as plasticizing agents reducing the glass transition temperature, Tg [35]. Polymerization reactions slow substantially when the Tg of the polymer/ monomer mixture reaches that of the surrounding due to solidification [36]. Solvents could therefore delay this solidification enabling conversion to continue for longer. These effects may have enabled both rapid and high final conversion with OS and OU in the presence of residual acetone. Longer term residual solvents, might leach out of the polymerized adhesive and be replaced by water. Of concern is this could weaken the adhesive and enable hydrolytic degradation of the adhesive interface [37,38].

Conversely, however, if viscosity is too low, the rapid acceleration in conversion that occurs at the gel point during polymerization reactions may not occur. This, in combination with the interaction between acetone and UDMA, might explain why conversion with acetone present in GB is limited. The optimal concentration of solvents for achieving maximum polymerization is typically higher than the concentration that leads to the best physical and mechanical properties of the adhesive resin. However, beyond a certain level, a higher solvent concentration can increase the distance between the radicals, which hinders the polymerization reaction [16,17]. The slow acetone evaporation rate and limited polymerization of GB in its presence is of clinical concern due to the likelihood of it resulting in the material properties being highly technique sensitive and potentially also failing to enable an effective bond between the composite and tooth structure.

## 5. Conclusion

The observation of similarities in difference spectra upon polymerization has enabled the development of a new method to determine the composition of dental adhesives during simultaneous drying and polymerization. Additionally, combined use of a reactant and product peak overcame difficulties in determining polymerization kinetics using standard methods that employ a reactant peak alone. Passive drying for 300s enabled acetone contents to reduce to 0% for OS and OU and 10% for GB. Conversely, without passive drying prior to light cure, acetone level decreased to final stable levels of 35, 20 and 32% for OS, OU and GB respectively. Whilst this loss was observed during 20s of light exposure with OS and OU, it occurred more slowly and after light exposure with GB. Passive drying more than doubled the rate of early monomer polymerization during light exposure, but post irradiation polymerization was reduced. Whilst final monomer conversions for GB increased from 40 to 77% with the passive drying step included, with OS and OU they decreased from 88 to 61% and 86–66% respectively. These results highlight the critical impact of handling conditions, such as drying time, on both chemical composition and polymerization outcome.

## Supporting information

S5 Data sheets raw data are in supplementary spreadsheets (XLSX)
